# Sol-Gel Synthesis, Spectroscopic and Thermal Behavior Study of SiO_2_/PEG Composites Containing Different Amount of Chlorogenic Acid

**DOI:** 10.3390/polym10060682

**Published:** 2018-06-19

**Authors:** Michelina Catauro, Elisabetta Tranquillo, Roberta Risoluti, Stefano Vecchio Ciprioti

**Affiliations:** 1 Department of Engineering, University of Campania “Luigi Vanvitelli”, Via Roma 29, I-81031 Aversa, Italy; elisabetta.tranquillo@unicampania.it; 2 Department of Biochemistry, Biophysics and General Pathology, Medical School, University of Campania “Luigi Vanvitelli”, Via L. De Crecchio 7, 80138 Naples, Italy; 3 Department of Chemistry—“Sapienza”, University of Rome, p.le A.Moro 5, 00185 Rome, Italy; roberta.risoluti@uniroma1.it; 4 Department S.B.A.I., Sapienza University of Rome, Via Del Castro Laurenziano 7, 00185 Rome, Italy; stefano.vecchio@uniroma1.it

**Keywords:** sol-gel, hybrids, chlorogenic acid, bioactivity, FTIR, TG

## Abstract

In this work, new phenol-based materials have been synthesized by the sol-gel method, in which different amounts of the phenolic antioxidant chlorogenic acid (CGA) (from 5 wt % to 20 wt %) were embedded in two different silica matrices: pure silica and silica-based hybrids materials, containing 50 wt % of polyethylene glycol (PEG). The incorporation of CGA in different sol-gel matrices might protect them from degradation, which could cause the loss of their properties. The two series of materials were chemically characterized by Fourier transform infrared (FTIR) spectroscopy. In addition, the thermal behavior of both series of materials containing CGA was studied by thermogravimetry under both air and inert N_2_ flowing gas atmosphere. The bioactivity was evaluated by soaking the synthesized hybrids in a simulated body fluid, showing that the bioactivity of the silica matrix is not modified by the presence of PEG and CGA.

## 1. Introduction

Biomaterial implants are clinically used in a variety of applications, and their interaction with cells or tissue is important for the patient health and quality of life. They should act as artificial replacements, since the primary requirement of any material is its biocompatibility inside the human body [1]. Immediately following implantation, proteins and other biomolecules that are present in the blood plasma and biological fluids rapidly adsorb on the surface of biomaterials, leading to structural changes and different biological response [2]. Adverse host responses to implanted biomedical devices could induce a cascade of biological events that cause implant failure [3], which is due to many factors, with infection and inflammations among the most important of them. Although inflammation recruits native cells for remodeling and regenerating the damaged tissue, persistent and inflammatory stimuli significantly interfere with the implant function, and often result in device failure [4]. During the inflammatory processes, the production of high amounts of reactive oxygen species (ROS) and H_2_O_2_ occurs. These inflammatory events of implant integration are necessary at the early stages for efficient wound healing, but a persistent oxidative stress might be the main cause of failure in orthopedic implants [5]. To overcome this problem, orally administered drugs have typically been used to control chronic tissue inflammation, but may not achieve adequate local concentrations. Therefore, controlled local release systems may provide the desired constant drug concentrations at the delivery site and a potential reduction of deleterious side effects [4,6].

In this work, new silica and silica/polyethylene glycol composites containing different percentages of chlorogenic acid, which is widely found in fruits and vegetables, were synthesized, characterized, and proposed as controlled drug delivery systems.

The synthesis procedure adopted was the sol-gel process, which is a technique widely used to make glasses and ceramics at low temperature. It is an interesting approach to prepare hybrid materials, because it takes place in solution, introducing easily the organic phases in an inorganic material, while preventing its degradation [7]. The reactions occurring during the sol-gel process are: (i) hydrolysis of alkoxide precursors to form a solution (sol phase) and (ii) condensation of the intermediate species that causes the formation of a 3D network (gel phase) [8]. This technique has been widely employed in the preparation of hybrid materials, composed by intimately distributed organic and inorganic phases.

It is reported in the literature that the sol-gel materials, in particular those with a silica matrix, have been investigated as carriers for different drugs [9,10]. In this work, two different organic compounds are incorporated in a silica matrix: polyethylene glycol (PEG) and chlorogenic acid (CGA). PEG is a versatile, biocompatible polymer that is mainly used in polymer-based materials for drug delivery [11]. It is recognized in many studies [12,13,14] that some of the most valuable biological properties of drugs, such as reducing toxicity and extending the circulation time of many drug nanocarriers, were improved by the addition of PEG into the material.

Chlorogenic acid is a member of the group of polyphenolic compounds that has many biological properties, including antibacterial, antihypertensive, antitumor, anti-inflammatory, and osteogenic potential [15,16,17,18,19,20]. The main purpose of incorporating CGA in the silica matrix is due to protecting the natural compound from adverse environmental conditions, such as the undesirable effects of light and oxygen, increasing the shelf life of the drug, and promoting a controlled delivery [21]. In particular, it is possible to protect the drug from degradation that causes the loss of its properties [22]. In fact, the decomposition products of CGA are widely recognized as undesirable compounds [23,24,25,26,27]. So, the knowledge of the decomposition behavior of CGA is extremely important in biomedical fields.

The aim of this study is the synthesis and characterization of SiO_2_/CGA and SiO_2_/PEG/CGA hybrid materials. Fourier transform infrared (FTIR) spectroscopy was used, similar to what has been made in recent studies for silica-based materials [28,29], to evaluate the interactions among different components incorporated in the hybrid materials. Furthermore, the hybrids were soaked in a simulated body fluid (SBF) to evaluate their ability to stimulate the hydroxyapatite nucleation by FTIR.

Thermal analysis is recognized as a powerful tool to analyze the thermooxidative behavior of polymer composites [30,31,32,33]. The thermal behavior of silica/CGA and silica/PEG/CGA materials under both air and inert N_2_ flowing gas atmosphere was studied by thermogravimetry under both inert and oxidative flowing gas atmosphere to analyze the different thermal behavior due to the inert or oxidative environments.

## 2. Materials and Methods 

### 2.1. Sol-Gel Synthesis

SiO_2_/CGA and SiO_2_/PEG/CGA organic–inorganic hybrids materials were synthesized by the sol-gel method according to a procedure reported in a recent study [34]. A solution of tetraethyl orthosilicate (TEOS, reagent grade, 98%, Sigma Aldrich, Steinheim am Albuch, Germany) was used as a precursor of the SiO_2_ inorganic matrix. TEOS, ethanol 99% (Sigma Aldrich, Steinheim am Albuch, Germany), nitric acid (solution 65%, Sigma Aldrich, Steinheim am Albuch, Germany), and water were added in a solution under stirring; nitric acid was used to promote the kinetics of hydrolysis and condensation reactions. The molar ratios among the reagents in the obtained solution are: EtOH/TEOS = 6.2, TEOS/HNO_3_ = 1.7, H_2_O/TEOS = 6. 

**Figure 1:**
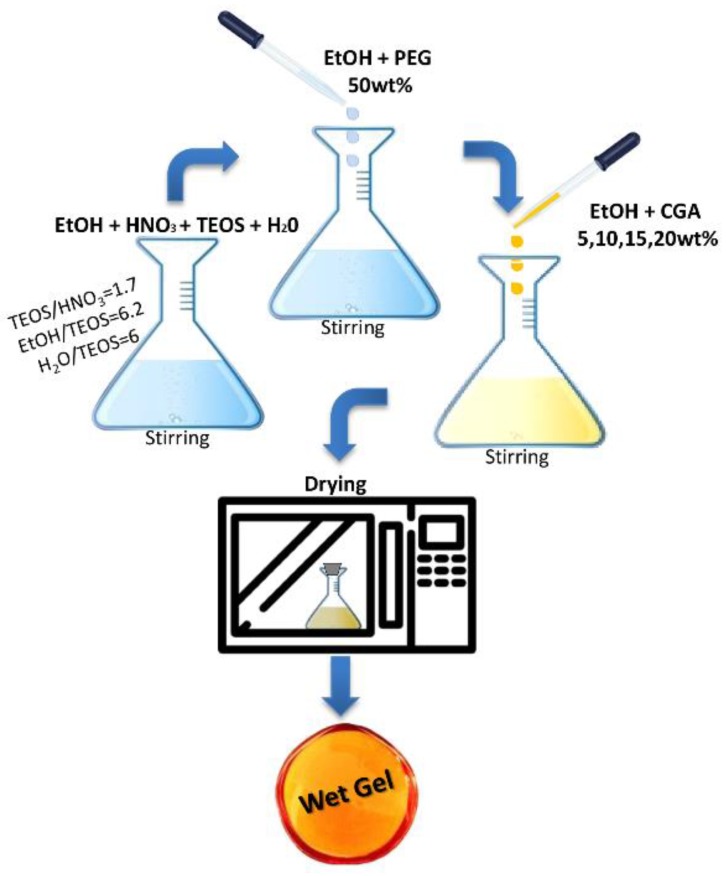
Flow chart of the sol-gel hybrid synthesis and the molar ratios between the reagents achieved in the sol.

The hybrid materials were obtained using ethanol solutions of CGA at different percentages (5 wt %, 10 wt %, 15 wt %, 20 wt %), while a high amount of polyethylene glycol (MW = 400, Sigma Aldrich) (PEG 50 wt %) dissolved in ethanol was added to the pure silica matrix (to reach 50 wt %) before preparing the SiO_2_/PEG/CGA materials. The different prepared solutions were put at room temperature until the gel was obtained, and then left into an oven at 40°C to allow for the removal of the residue solvent, thus avoiding the thermal degradation of the drug. The flow chart of the sol-gel process is shown in Figure 1.

### 2.2. Materials Characterization

FTIR spectroscopy was used to investigate the chemical composition of the materials and the interactions among their components. A Prestige 21 (Shimadzu, Kyoto, Japan) system, equipped with a DTGS KBr (deuterated triglycine sulfate with potassium bromide windows) detector allowed us to record transmittance spectra in the 400–4000 cm^−1^ region, with a resolution of 2 cm^−1^ (45 scans). Then, 2 mg of sample powder, mixed with 198 mg of KBr, was compacted into discs under a pressure of 7 tons by using a hydraulic press (Specac, Orpington, UK). The FTIR spectra were elaborated by the Prestige software (IR solution).

Thermogravimetric (TG) analysis of all of the investigated samples was performed by a TGA7 Thermobalance (PerkinElmer, Waltham, MA, USA). Samples were placed into a platinum crucible, and the temperature was measured using a thermocouple directly connected to the crucible. The temperature was raised from 20 °C to 800 °C at a heating rate of 10 °C min^−1^, with the best resolution rate permitting to differentiate the contribution of all of the components. The TG analyses were performed both under an inert gas flux of nitrogen and oxidant atmosphere of air at 100 mL min^−1^. Temperature calibration was performed using the Curie-point transition of standard metals, as specified by the equipment recommendations. Each sample was analyzed in triplicate, and a high reproducibility of the resulting curves was observed.

### 2.3. Bioactivity Test

The in vitro apatite-forming ability test [35] was used to evaluate the bioactivity of the hybrid materials.

All of the hybrid materials were crushed to a powder using an agate mortar and soaked for seven, 14 and 21 days in a simulated body fluid (SBF) with ion concentrations nearly equal to those found in human blood plasma. In order to maintain the SBF solution temperature fixed at 37 °C, the samples were placed in polystyrene bottles in a water bath. As the ratio between the total surface area of the material exposed to SBF and its volume influences the reaction of hydroxyapatite nucleation, a constant ratio was maintained, as reported in the literature. Moreover, the SBF solution, in which the samples were soaked, was exchanged every two days to avoid depletion of the ionic species in the SBF due to the formation of biominerals. After each soaking period, the samples were removed from the SBF and air-dried in a desiccator. After 24 h, they were subjected to FTIR analysis in order to evaluate the ability to form an apatite layer on their surfaces.

## 3. Results and Discussion

### 3.1. FTIR Structural Characterization

**Figure 2:**
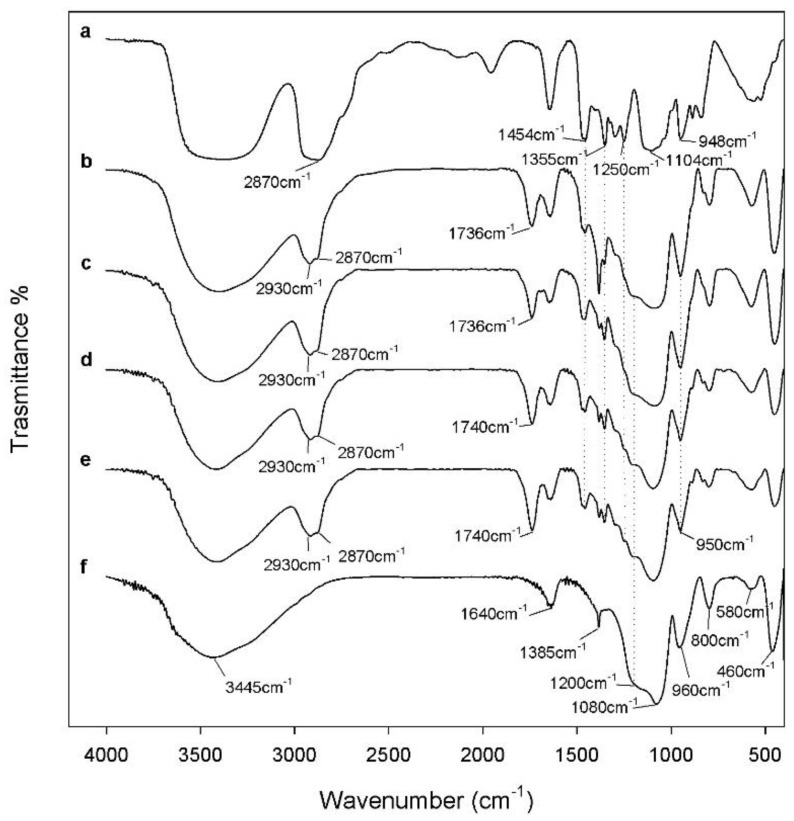
Representative Fourier transform infrared (FTIR) spectra of: (**a**) pure polyethylene glycol (PEG); (**b**) SiO_2_/PEG_50 wt %_/chlorogenic acid (CGA)_5 wt %_; (**c**) SiO_2_/PEG_50 wt %_/CGA_10 wt %_; (**d**) SiO_2_/PEG_50 wt %_/CGA_15 wt %_; (**e**) SiO_2_/PEG_50 wt %_/CGA_20 wt %_; (**f**) pure SiO_2_.

In order to evaluate the chemical structure of SiO_2_/PEG/CGA hybrid materials, their FTIR spectra are displayed in Figure 2. The hybrids’ spectra of materials were compared with pure PEG and pure silica to identify their interaction. In the SiO_2_ spectrum (curve f), all of the typical peaks of the silica sol-gel materials [36,37] are visible. In particular, the asymmetric and symmetric Si–O stretching vibrations are recorded at 1080 cm^−1^ with shoulders at 1200 cm^−1^ and at 800 cm^−1^. The peaks at 460 cm^−1^ and 960 cm^−1^ were attributed to the bending vibrations of Si–O–Si bonds and Si–OH bond vibrations, respectively [38]. Furthermore, in the pure silica spectra, it is possible to observe the low intensity band at 580 cm^−1^ due to residual four-membered siloxane rings in the silica network [36,37,39,40] and the sharp N–O stretching band of residual nitrate anions at 1382 cm^−1^ [41] caused by the presence of residual HNO_3_, which was used as a catalyst in the synthesis procedure. The position and shape of the bands at 3445 cm^−1^ and 1640 cm^−1^ attributed to –OH stretching and bending vibrations in the hydration water suggest the presence of H-bonded solvent molecules (H_2_O) and hydrogen bonded –OH groups attached to the Si atoms. The addition of high amounts of PEG (50%) in the silica matrix allows observing, in the hybrid spectra (curve b–e), some peaks typical of the polymer. The bands at 2930–2870 cm^−1^ and 1454 cm^−1^ are due to PEG methylene C–H stretching and bending, respectively [42]. The typical signals of the C–C groups at 948 cm^−1^ are clearly visible, as well as the characteristic C–O stretching band of alcohols at 1250 cm^−1^ [43,44]. In the hybrid spectra, the change in the shape of the broad band at about 3400 cm^−1^, as well as the displacement of the Si–OH band and the peak at 580 cm^−1^ at a lower wavenumber, may be due to the formation of hydrogen bonds [45] between the –OH groups of the inorganic phases and ethereal oxygen atoms (H-bond donors) or terminal –OH in the PEG chains. These results suggest that the presence of chlorogenic acid does not change the structure and interactions of SiO_2_/PEG hybrid materials.

**Figure 3:**
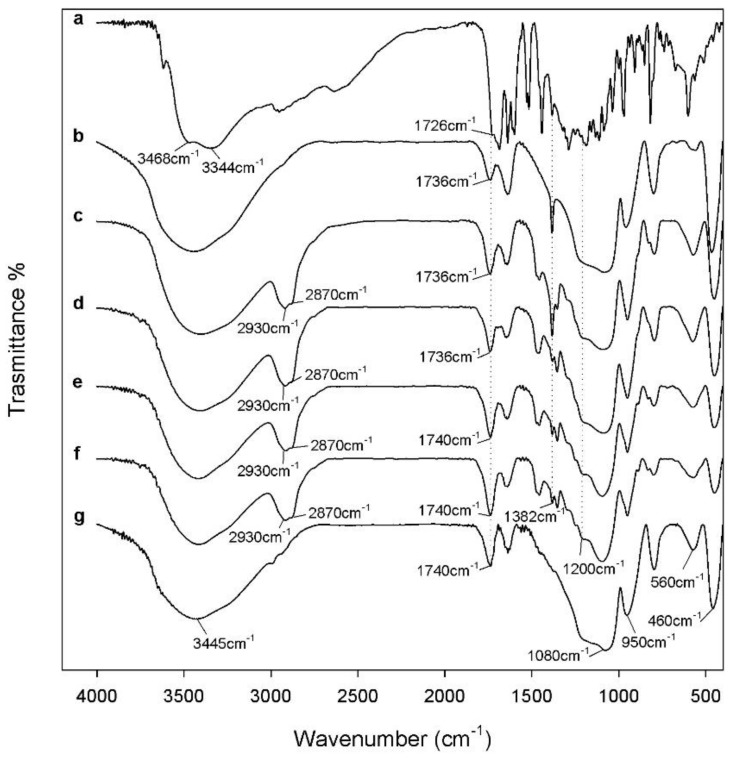
Representative FTIR spectra of: (**a**) pure CGA; (**b**) SiO_2_/CGA_5 wt %_; (**c**) SiO_2_/PEG_50 wt %_/CGA_5 wt %_; (**d**) SiO_2_/PEG_50 wt %_/CGA_10 wt %_; (**e**) SiO_2_/PEG_50 wt %_/CGA_15 wt %_; (**f**) SiO_2_/PEG_50 wt %_/CGA_20 wt %_; (**g**) SiO_2_/CGA_20 wt %_.

Figure 3 shows the FTIR spectra of the hybrids with different percentages of CGA_(5,10,15,20 wt %)_ compared with pure CGA (curve a), SiO_2_/CGA_5 wt %_, and SiO_2_/CGA_20 wt %_ (curves b, g). The spectrum of pure CGA shows the stretching of OH groups at 3468 cm^−1^ and 3344 cm^−1^, whereas the OH bending of the phenol function is visible at 1382 cm^−1^ [34]. The latter band in the spectrum of SiO_2_/PEG_50 wt %_/CGA_5 wt %_ hybrid (curve c) is more intense than that related to the other hybrids containing the polymer. In particular, this peak (1382 cm^−1^) is visible also in the spectrum of SiO_2_/CGA_5 wt %_ (curve b), which is probably due to an interaction between the silica matrix and CGA: increasing the drug concentration caused a decrease of the peak intensity, because the drug at low concentrations is more embedded and linked within it by the formation of –H bonds. On the other hand, when a higher amount of CGA is added in pure silica (curve g) and in the silica/PEG matrix (curves d, e, f), a part of CGA cannot form H-bonds, because all of the hydroxyl groups of silica are already involved in H-bonds.

The stretching C=O vibration band is observed at 1726 cm^−1^ in the spectrum of pure CGA; instead, its displacement occurs in all of the hybrids’ spectra, this effect can be explained by the establishment of H-bonds with the SiO_2_ inorganic matrix [34]. The different shape and a broadening of the SiO_2_ strong band at 1080 cm^−1^, as well as a marked increase of the intensity of the shoulder at 1200 cm^−1^, is due to the presence in this spectral region of intense signals of the phenyl ring and C–O–C bonds in CGA (see curve a), and also to interactions with the polymer. 

Therefore, all of the acquired spectra suggest that chlorogenic acid was embedded in the silica/PEG matrix, and all of the components are linked to each other by hydrogen bonds, which play a crucial role in the structure of hybrids.

### 3.2. Thermal Behavior Study

**Figure 4:**
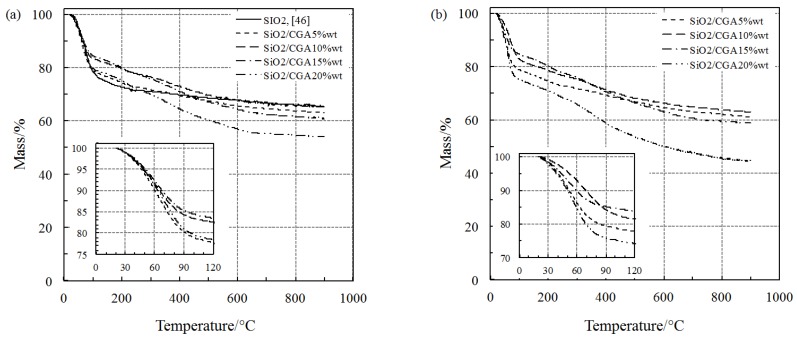
Thermogravimetric (TG) curves of pure SiO_2_ and SiO_2_/CGA hybrids at 10 °C min^−1^ under a flowing atmosphere of: nitrogen (**a**); air (**b**).

**Figure 5:**
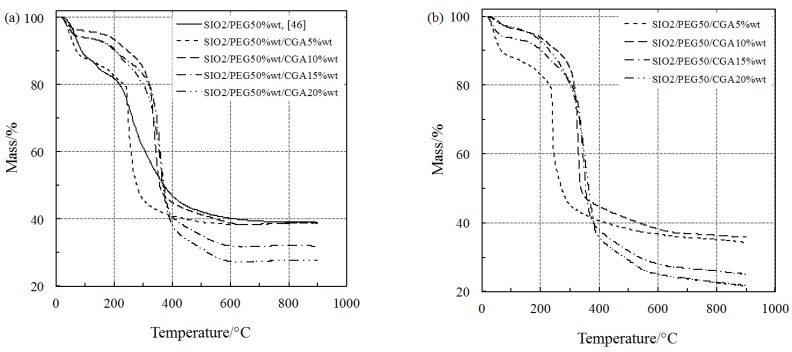
TG curves of SiO_2_/PEG and SiO_2_/PEG/CGA hybrids at 10 °C min^−1^ under a flowing atmosphere of: nitrogen (**a**); air (**b**).

The thermal behavior of the two classes of hybrids under inert and oxidative atmosphere has been studied by recording TG experiments in flowing nitrogen and air at 10 °C min^−1^ (see the Materials and Methods Section). The corresponding TG curves of the SiO_2_/CGA and SiO_2_/PEG_50 wt %_/CGA materials (with increasing content of CGA from 5 wt % to 20 wt %) are reported in Figure 4 and Figure 5, respectively. For comparison purposes, the TG curves of both pure silica and SiO_2_/PEG_50 wt %_ carried out under a stream of Ar and recently published [46] are also reported in both plots *a* of Figure 4 and Figure 5, respectively.

Under inert atmosphere, the SiO_2_/CGA hybrids undergo a first mass loss of up to 120 °C (Figure 4a) ascribed to dehydration, although the loss of residual ethanol used for the synthesis cannot be excluded. The thermal behavior of these materials in this temperature range is identical to that of pure silica [46], as is evident by the perfect superimposition of the TG curves. The water released by the CGA-poorest and CGA-richest hybrids (SiO_2_/CGA_5 wt %_ and SiO_2_/CGA_20 wt %_, respectively) when this process reaches its completion from 100 °C to 120 °C (see the inner plot of Figure 4a) is about 9% higher than that of the other two. A high amount of weakly bonded water is removed from SiO_2_/CGA_5 wt %_, since only a limited number of H-bonds is formed because of the poor content of CGA. On the other hand, SiO_2_/CGA_20 wt %_ may retain a remarkable content of water and/or ethanol (removed by dehydration in the first step of the TG curve), because the higher the amount of CGA in the hybrid, the higher the number of formed H-bonds. Slight differences in the percentages of mass loss due to dehydration were observed when the same process takes place in air (Figure 4b), where the temperature range for dehydration shifts toward lower values as the amount of water increases (inner plot in Figure 4b). 

Starting from about 160 °C, a second step of mass loss occurred in a wide temperature range in both inert and air atmosphere for all of the SiO_2_/CGA hybrids. This loss was probably ascribable to the thermal degradation (pyrolysis) of CGA (plot of both Figure 4a,b, respectively) while pure SiO_2_ undergoes dehydroxylation [46]. It also corresponds to the slow elimination of water in a wide temperature range, which is caused by the condensation of surface hydroxyl groups. 

As far as the multi-step thermal degradation of CGA is concerned, higher percentages of mass were recorded for materials treated in air with respect to those in inert atmosphere, even though the shapes of the curves seem to be quite similar. This different thermal behavior of the materials on the basis of the different atmosphere of nitrogen and air is substantially the same as that observed in a recent study concerning the multi-step thermal degradation of pure CGA [15]. The thermal behavior of all of the SiO_2_/PEG/CGA materials under nitrogen atmosphere is shown in Figure 5a. The poor-CGA material (SiO_2_/PEG_50 wt %_/CGA_5 wt %_) shows a TG profile similar to that of SiO_2_/PEG_50 wt %_ [46]: the water released is remarkably higher than those of the other SiO_2_/PEG/CGA materials, but is comparable with that of SiO_2_/PEG_50 wt %_, while the dehydration temperature is slightly shifted toward lower values (by a few degrees). The other SiO_2_/PEG/CGA materials (with different amounts of CGA ranging from 10 wt % to 20 wt %) showed a loss of water between 3% and 6%. At higher temperatures, they undergo consecutive decomposition processes of CGA and PEG up to 600 °C. In particular, the thermal decomposition of CGA seems to take place around 200 °C for SiO_2_/PEG_50 wt %_/CGA_5 wt %_, followed by that of PEG, which is sharper than that for SiO_2_/PEG_50 wt %_ [46] (see Figure 5). It can be concluded that the presence of CGA does not significantly affect the thermal behavior of materials containing both SiO_2_ and PEG. 

Unexpectedly, the oxidation environment seems not to influence the thermal behavior of these materials with respect to the inert one (air with respect to nitrogen, respectively), as it is clearly observed by comparing the TG curves in Figure 5b with those in Figure 5a (they are almost superimposable).

### 3.3. Bioactivity Test

**Figure 6:**
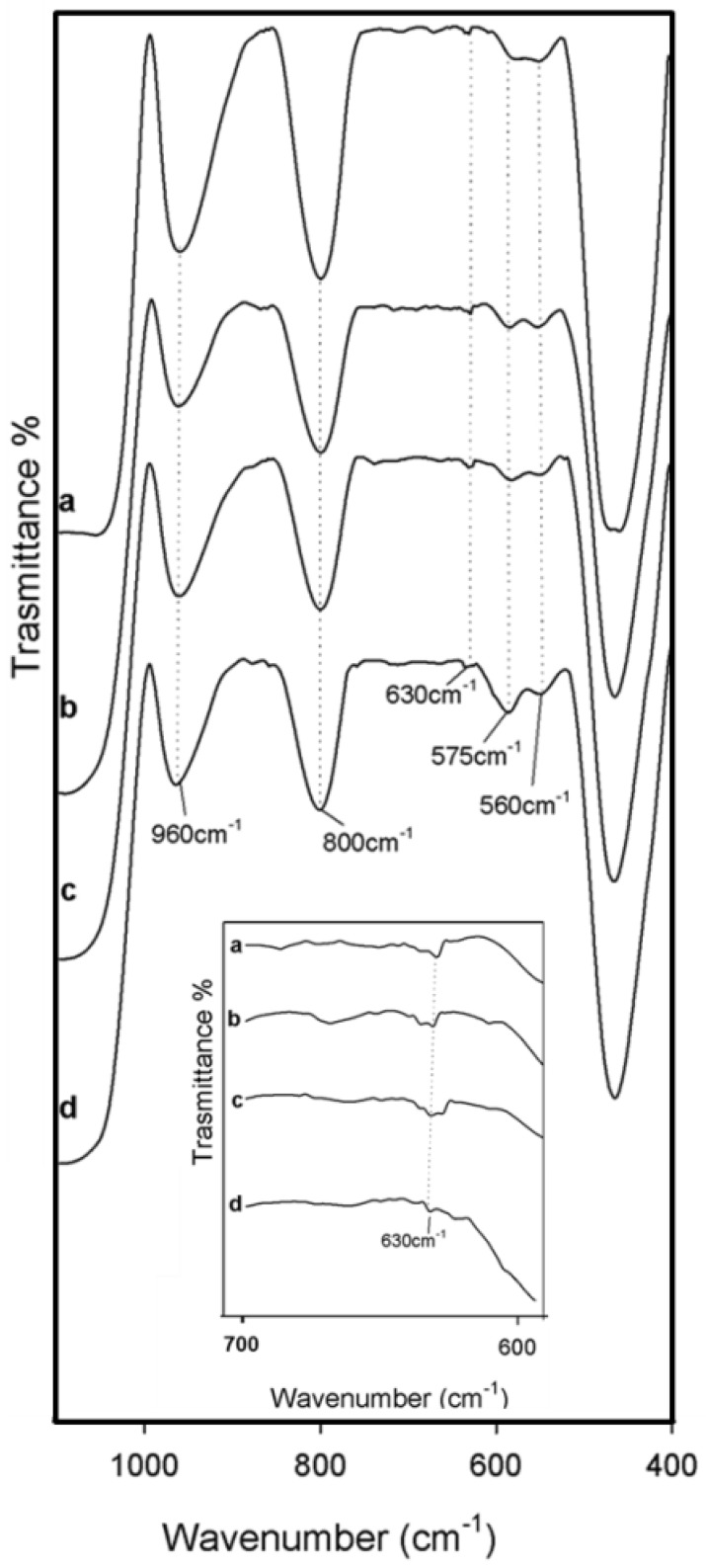
Representative FTIR spectra of (**a**) SiO_2_/PEG_50 wt %_/CGA_5 wt %_; (**b**) SiO_2_/PEG_50 wt %_/CGA_10 wt %_; (**c**) SiO_2_/PEG_50 wt %_/CGA_15 wt %_; (**d**) SiO_2_/PEG_50 wt %_/CGA_20 wt %_ after 21 days of exposure to SBF.

The bioactivity of hybrid materials was evaluated by a Kokubo test [35]. FTIR analysis was used to detect the nucleation of hydroxyapatite on the surfaces of all of the samples after 21 days in SBF. Figure 6 shows the FTIR spectra of the hybrids, in which a new band at 630 cm^−1^ (inner plot in Figure 6) and the split of that at 570 cm^−1^ in two new ones at 575 cm^−1^ and 560 cm^−1^, respectively, were observed. These bands are due to the stretching of the –OH groups of hydroxyapatite and the vibrations of the P$O_{4}^{3-}$ groups caused by the formation of the hydroxyapatite precipitate. Furthermore, the interaction of the hydroxyapatite layer with the –OH groups of the silica matrix was suggested by the displacement of Si–OH band, from 955 cm^−1^ to 960 cm^−1^. The presence of Si–OH groups on the surface of the hybrids stimulates hydroxyapatite nucleation, because these groups attract the Ca^2+^ ions present in the fluid, thus leading to an increase of the positive surface charge. The Ca^2+^ ions combine with the negative charge of the phosphate ions to form amorphous phosphate, which spontaneously transforms into hydroxyapatite [Ca_10_(PO_4_)_6_(OH)_2_] [47].

## 4. Conclusions

In this study, new hybrid materials with different percentages of CGA and PEG were prepared by sol-gel synthesis. FTIR analysis demonstrated that hydrogen bonds occurred in all of the materials between the inorganic silica matrix and the organic components. This result suggests that both the polymer and CGA are embedded in the matrix and interact with it. 

The thermal behavior of the two different hybrid materials has been studied in both inert and oxidative atmosphere. The TG curves show that at higher temperatures, the materials undergo consecutive decomposition processes of CGA and polymer up to 600 °C. The oxidation environment seems not to influence the thermal behavior of these materials with respect to the inert one.

Furthermore, the materials were soaked in a simulated body fluid to evaluate their bioactivity. The FTIR spectra showed also the formation of a hydroxyapatite precipitate on the sample surfaces, thus proving that the presence of both organic components does not affect the bioactivity of the silica matrix nor the thermal behavior.
